# Combined effects of blood flow restriction training and nutritional intervention on muscle adaptations: a systematic review and meta-analysis

**DOI:** 10.3389/fnut.2026.1762391

**Published:** 2026-05-08

**Authors:** Bingran Zhao, Haiting Zhai

**Affiliations:** 1 Sports Coaching College, Beijing Sports University, Beijing, China; 2 Naval Aviation University, Yantai, China

**Keywords:** blood flow restriction training, muscle hypertrophy, muscle strength, muscular endurance, nutritional supplementation

## Abstract

**Background:**

Blood flow restriction (BFR) training induces muscle hypertrophy and strength gains under low-load conditions by restricting blood flow. While BFR reduces mechanical stress on joints, the associated metabolic stress and discomfort may limit training performance. Certain nutritional supplements may enhance BFR training effects, but the existing evidence remains inconclusive.

**Objective:**

To systematically evaluate the combined effects of BFR training and nutritional interventions on maximal strength, muscular endurance, and muscle hypertrophy in healthy adults.

**Methods:**

A systematic search was conducted in PubMed, Web of Science, Embase, Cochrane Library, and Scopus databases from January 2015 to December 2025, following PRISMA guidelines. Randomized controlled trials comparing BFR combined with nutritional interventions versus BFR alone (or BFR plus placebo) were included. Standardized mean differences (SMD) and 95% confidence intervals (CIs) were calculated using a random-effects model.

**Results:**

Nine studies involving 181 healthy adults were included. Meta-analysis showed no clear additional effect of nutritional interventions on maximal strength (SMD = −0.09; 95% CI: −0.37, 0.20; *p* = 0.55) or muscle hypertrophy (SMD = 0.31; 95% CI: −0.14, 0.77; *p* = 0.18). Nutritional interventions were associated with improvements in muscular endurance (SMD = 0.90; 95% CI: 0.55, 1.25; *p* < 0.00001), although the limited number of studies and small sample sizes warrant cautious interpretation. Subgroup analyses indicated no significant influence of supplement type, participant training status, or intervention duration on maximal strength.

**Conclusion:**

Current evidence suggests that nutritional interventions may support fatigue resistance and enhance muscular endurance under BFR training conditions, but do not provide a clear additional benefit for maximal strength or hypertrophy. These findings should be interpreted cautiously due to the limited number of studies, small sample sizes, and heterogeneity in supplementation protocols. Further high-quality research is needed to explore long-term effects, mechanisms, and population-specific responses, including female and elderly participants.

## 1 Introduction

Blood flow restriction (BFR) training, originally developed through early KAATSU- and vascular occlusion-based resistance training paradigms in the early 2000s, has gained increasing attention as a low-load training strategy in sports science and rehabilitation medicine (1–3, 41–43). Its core mechanism involves the application of external pressure (e.g., cuffs) at the proximal limbs to restrict venous return and partially occlude arterial blood flow, thereby inducing muscle hypertrophy and strength gains comparable to those achieved with traditional high-load resistance training, even under low-load conditions (typically 20–30% 1RM) (4–6). This training approach not only reduces mechanical stress on joints and soft tissues but also induces substantial local metabolic stress by promoting hypoxia, acidosis, and metabolite accumulation, which recruits high-threshold motor units and stimulates anabolic signaling (1, 3, 6). Consequently, BFR has been increasingly applied in older adults and musculoskeletal rehabilitation settings, including post-surgical populations, and has also been used in competitive athletes seeking to overcome training plateaus (1, 2, 7, 44). In addition, BFR has been explored in selected clinical populations, such as women with rheumatoid arthritis, suggesting its broader applicability under low-load conditions (45, 46).

However, the physiological conditions inherent to BFR training also present certain limitations. The metabolically challenging environment induced by BFR, characterized by the accumulation of metabolites such as lactate and hydrogen ions, not only causes localized pain and subjective discomfort but may also lead to premature fatigue, thereby hindering phosphocreatine resynthesis and limiting further improvements in training volume and quality (8–10). To overcome these limitations and maximize training adaptations, researchers have begun to explore combining BFR training with specific nutritional interventions. Theoretically, certain supplements may exert synergistic effects with BFR through targeted physiological mechanisms. For example, creatine may alleviate phosphagen depletion (11), *β*-alanine may counteract acidosis (12), nitrate-based vasodilators may improve microcirculatory oxygenation (13, 14), and caffeine may reduce pain perception through central regulation (9, 15). Despite the theoretical appeal of this combined strategy, empirical evidence remains inconsistent. On the one hand, some studies suggest that protein or creatine supplementation does not further enhance gains in maximal strength or muscle cross-sectional area induced by BFR training, indicating that BFR itself may already produce a strong adaptive stimulus (7, 16, 17). On the other hand, several studies have reported significant gains in muscular endurance and related performance outcomes (9, 11, 14). Furthermore, no systematic quantitative synthesis has yet clarified whether supplement type and participants’ training status modify these interactive effects.

Given these inconsistencies, the aim of the present study was to systematically assess the effects of combining BFR training with nutritional interventions on maximal strength, muscular endurance, and muscle morphology in healthy adults through a systematic review with meta-analysis. This study seeks to clarify the specific application value of nutritional supplements within the BFR training framework and explore the underlying physiological mechanisms, thereby providing evidence-based recommendations for the development of more precise, personalized training and nutrition strategies.

## 2 Methods

This systematic review with meta-analysis was conducted in accordance with the Preferred Reporting Items for Systematic Reviews and Meta-Analyses (PRISMA) guidelines. Two authors independently screened and assessed eligible studies, and any disagreements were resolved through discussion or consultation with a third author. The review protocol was prospectively registered in PROSPERO in November 2025 (CRD420251176839).

### 2.1 Literature search strategy

Studies were identified through systematic searches of PubMed, Web of Science, the Cochrane Library, Embase, and Scopus from January 1, 2015 to December 31, 2025. The final search was completed in December 2025. Reference lists of included studies were also screened to identify additional eligible articles.

Search strategies were adapted for each database using controlled vocabulary terms (where applicable) and free-text keywords. The search terms covered three main domains: blood flow restriction training, nutritional interventions, and the prespecified outcome domains (maximal strength, muscular endurance, and muscle morphology/hypertrophy). Boolean operators, phrase searching, and truncation were applied as appropriate for each database. Searches were limited to titles, abstracts, and keywords, and only studies published in English were considered. Full search strategies for all databases are provided in Supplementary Table S1.

### 2.2 Inclusion criteria

The inclusion criteria were defined according to the PICOS framework: (1) healthy adults aged ≥18 years with a body mass index (BMI) between 18 and 30 kg/m^2^; (2) participants without cardiovascular, metabolic, renal, or other major medical conditions; (3) interventions involving both blood flow restriction (BFR) training and a concurrent nutritional intervention; (4) BFR pressure set at 40–80% of arterial occlusion pressure (AOP) or an absolute pressure of 100–200 mmHg; (5) nutritional interventions including macronutrient manipulation, functional supplements such as creatine or caffeine, or specific dietary strategies performed concurrently with BFR training; (6) randomized controlled trials (RCTs); and (7) studies reporting at least one objective outcome related to maximal strength, muscular endurance, or muscle morphology/hypertrophy, such as 1RM, maximal voluntary contraction (MVC), repetitions to failure, time to fatigue, muscle cross-sectional area, muscle thickness, or fat-free mass.

### 2.3 Exclusion criteria

**Figure 1:**
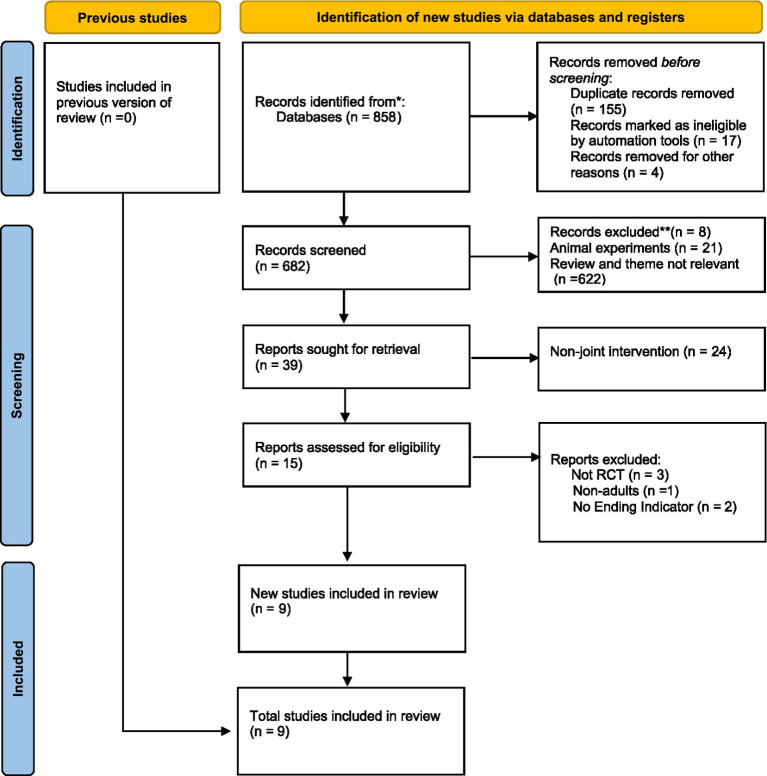
Flow diagram.

Studies were excluded if they: (1) included participants younger than 18 years, pregnant women, or individuals with clinical conditions such as diabetes, kidney disease, or cardiovascular events; (2) did not quantify the nutritional intervention or did not administer it concurrently with BFR training; (3) combined BFR with other interventions such as pharmacological treatment, electrical stimulation, hyperbaric oxygen therapy, or hypoxic training, which could confound the independent effects of BFR combined with nutritional interventions; (4) reported only subjective outcomes (e.g., rating of perceived exertion [RPE] or visual analogue scale [VAS]) without any objective measures of strength, endurance, or muscle morphology; or (5) were conference abstracts, reviews, theses, editorials, or methodological papers. The study selection process is presented in Figure 1 in the Results section.

### 2.4 Data extraction

Data were independently extracted by two researchers (B.R.Z. and H.T.Z.) using a standardized, predesigned form. Extracted information included study characteristics, participant characteristics, intervention details, and outcome measures. When results were reported as standard errors (SEs) or confidence intervals (CIs), they were converted to standard deviations (SDs) using standard formulas. When numerical values were presented only graphically, data were extracted using WebPlotDigitizer. Any discrepancies were resolved through discussion or consultation with a third researcher.

### 2.5 Risk of Bias assessment

The risk of bias of the included randomized controlled trials was independently assessed by two researchers (B.R.Z. and H.T.Z.) using the Cochrane Risk of Bias tool (18). The following domains were evaluated: random sequence generation, allocation concealment, blinding of participants and personnel, blinding of outcome assessment, incomplete outcome data, selective reporting, and other potential sources of bias. Each domain was rated as “low risk,” “high risk,” or “unclear risk.” Disagreements were resolved through discussion with a third researcher. The overall risk-of-bias profile was considered when interpreting the certainty and robustness of the pooled findings.

### 2.6 Data synthesis and statistical analysis

All statistical analyses were performed using Review Manager software (RevMan version 5.4, The Cochrane Collaboration, Oxford, UK). Because methodological heterogeneity was expected due to differences in supplementation protocols, BFR procedures, training duration, and outcome measurements across studies, a random-effects model was applied (19). Pooled effects were expressed as standardized mean differences (SMDs) with 95% confidence intervals (CIs).

Effect sizes were interpreted according to Hopkins’ criteria: <0.20 as trivial, 0.20–0.60 as small, 0.61–1.20 as moderate, 1.21–2.00 as large, and >2.00 as very large (20). Statistical heterogeneity was assessed using the *I*^2^ statistic (21), with values <25, 25–75%, and >75% indicating low, moderate, and high heterogeneity, respectively. Statistical significance was set at *p* < 0.05.

Sensitivity analyses were conducted by sequentially removing individual studies to examine the robustness of the pooled estimates. Given the small number of included studies for several outcomes, the meta-analysis may have been underpowered to detect small effects, and the pooled findings should therefore be interpreted cautiously. Funnel plots were visually inspected as an exploratory assessment of publication bias. However, because fewer than 10 studies were available for each pooled outcome, funnel plots were considered unreliable for formally excluding publication bias (22).

## 3 Results

### 3.1 Identification and characteristics of the included studies

A total of nine studies were included in the analysis (7, 9, 11–15, 17, 23), involving a total of 181 participants. The study selection process is presented in Figure 1. As shown in Table 1, the participants were primarily healthy untrained individuals, recreationally active individuals, and trained participants, with a BMI range of 21.9–27.0 kg/m^2^.

**Table 1:** Characteristics of included studies.

First author (year)	Country	Participants (N) [Exp/Ctrl]	Age (y)/training status	BFR Training protocol	Nutrition intervention	Dietary control/notes	Outcomes measured
Lin et al. (15)	Taiwan	14/14	23.0 ± 2.0/Untrained	Wrist extension; 20% MVC; 60% SBP; 4 sets (30–15–15-15)	Caffeine: 6 mg/kg, 1 h pre-exercise	Abstained from caffeine/alcohol for 24 h	MVC, Force fluctuations
Vendruscolo et al. (17)	Brazil	13/8	26 ± 6/Trained (>1 yr)	Biceps curl; 30% 1RM; 80% AOP; 4 sets x 15 reps	Whey Protein: 25 g × 2/day (Post-ex and Pre-sleep)	3-day food record; Maintained habitual diet	1RM, CSA, RPE, Pain
Sousa-Silva et al. (11)	Brazil	8/9	23.8 ± 3.7/Untrained	Biceps curl; 30% 1RM; 50% AOP; 4 sets (30–15–15–15)	Creatine: Load 20 g/d × 5d; Maint 5 g/d × 8w	3-day food record; Double-blind	1RM, Muscular Endurance, FFM
Pessoa et al. (2023)	Brazil	9/10	21.9 ± 2.8/Untrained	Biceps curl; 30% 1RM; 50% AOP; 3 sets (30–15–15)	beta-Alanine: 4.8 g/d (sustained-release); divided doses	3-day food record; Double-blind	1RM
Machek et al. (23)	USA	9/9	25 ± 5/Rec. Trained	Unilateral leg press; 20% 1RM; 80% AOP; 4 sets + 2 failure sets	Betaine: 6 g/d (3 g x 2); 14 days loading	24-h dietary recall; Double-blind	Reps to failure, RPE, Pain, Hormones
Centner et al. (7)	Germany	11/11	61.7 ± 5.5/Untrained	Leg press; 20–30% 1RM; 50% AOP; 4 sets (30–15–15-15)	Collagen Hydrolysate: 15 g/d; Within 60 min post-ex	3-day food record; Double-blind	1RM (% change), CSA (% change)
Souza et al. (9)	Brazil	11/11	23.4 ± 4.1/Trained (>2 years)	Unilateral knee extension; 30% 1RM; 80% AOP; 3 sets to failure	Caffeine: 6 mg/kg; 1 h pre-test	Low habitual caffeine intake	Total Repetitions, RPE, Pain, Lactate
Papadopoulos et al. (14)	Greece	16/16	21.9 ± 0.5 /Mod. Active	Handgrip; 30% MVC; Arterial Occlusion (250 mmHg); Sustained to failure	Nitrate (Beetroot Juice): 140 mL (8.1 mmol NO3-); 2.5 h pre-test	Avoided high-nitrate diet for 48 h	MVC, Time to fatigue
Hoon et al. (13)	Australia	18/18	29 ± 6/Healthy Active	Knee extension; Electrical stimulation; 250 mmHg occlusion; Fatigue protocol	Nitrate (Beetroot Juice): 525 mg NO3−/d x 3d; 1,050 mg on test day	48-h dietary record; Double-blind	MVC, Fatigue resistance

RCT, Randomized Controlled Trial; Exp/Ctrl, Experimental Group/Control Group; M/F, Male/Female; BFR, Blood Flow Restriction; MVC, Maximal Voluntary Contraction; 1RM, One-Repetition Maximum; AOP, Arterial Occlusion Pressure; SBP, Systolic Blood Pressure; CSA, Cross-Sectional Area; FFM, Fat-Free Mass; RPE, Rating of Perceived Exertion.

The parameters of BFR combined with nutritional intervention varied across studies. BFR cuff pressure ranged from 40 to 80% of arterial occlusion pressure (AOP) or was applied as an absolute pressure of 100–250 mmHg. Nutritional interventions included caffeine (9, 15), creatine (11), *β*-alanine (12), collagen (7), whey protein (17), and betaine and nitrate supplementation (13, 14, 23). Intervention duration ranged from acute single-dose protocols (9, 13, 14) to chronic supplementation protocols lasting 2–8 weeks (7, 11, 12, 15, 17, 23).

**Figure 2:**
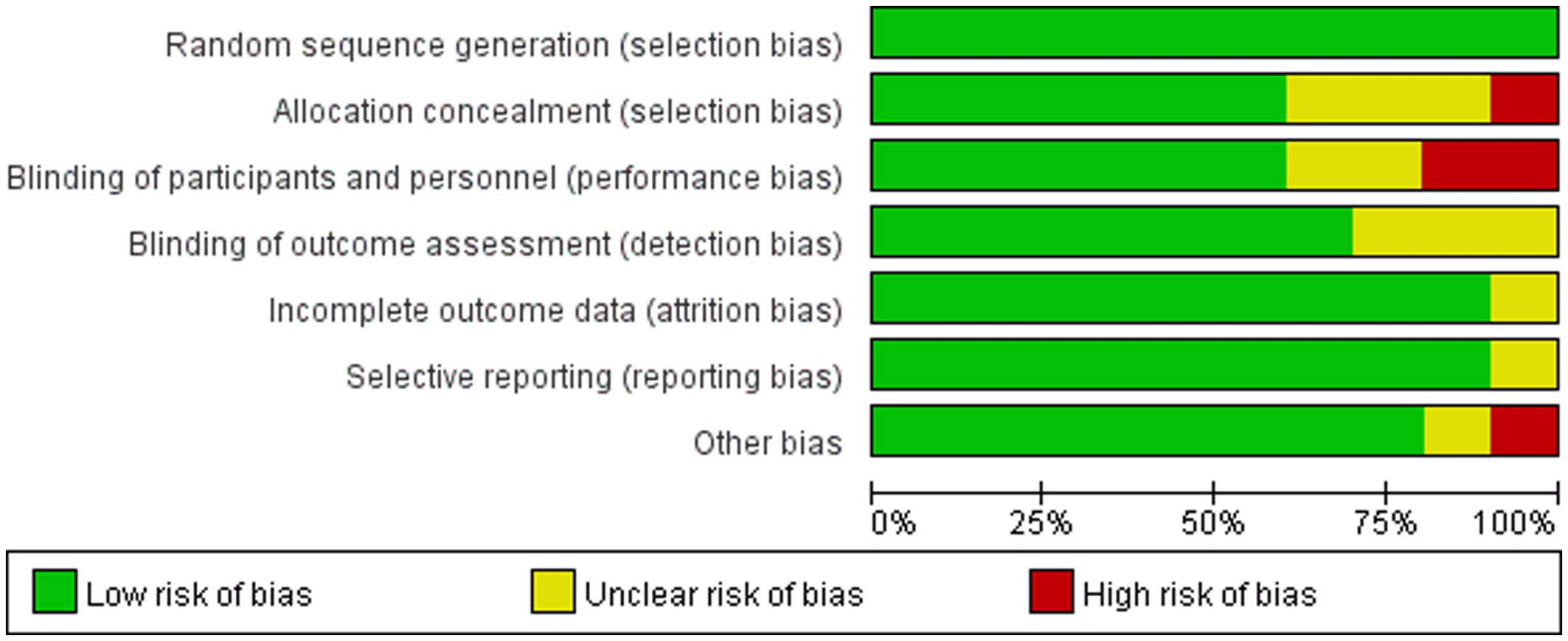
Risk of bias graph.

**Figure 3:**
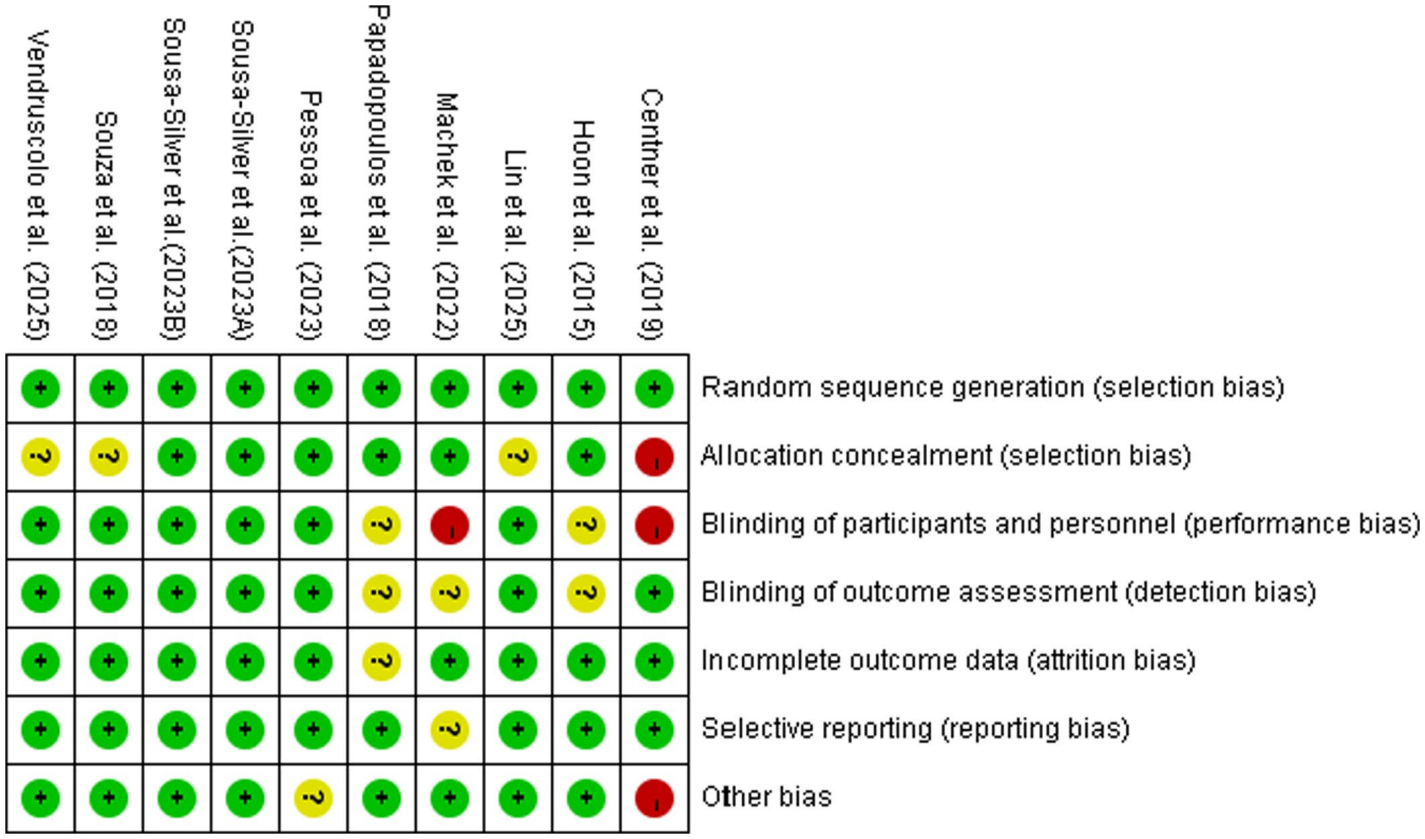
Risk of bias summary.

The risk-of-bias assessment is summarized in Figures 2, 3. Overall, most studies were judged as having low or unclear risk across the assessed domains, with relatively favorable ratings for random sequence generation, outcome assessment, and completeness of outcome data. However, some domains remained insufficiently reported in several studies, and these considerations were taken into account when interpreting the pooled findings.

### 3.2 Overall effect analysis

**Figure 4:**
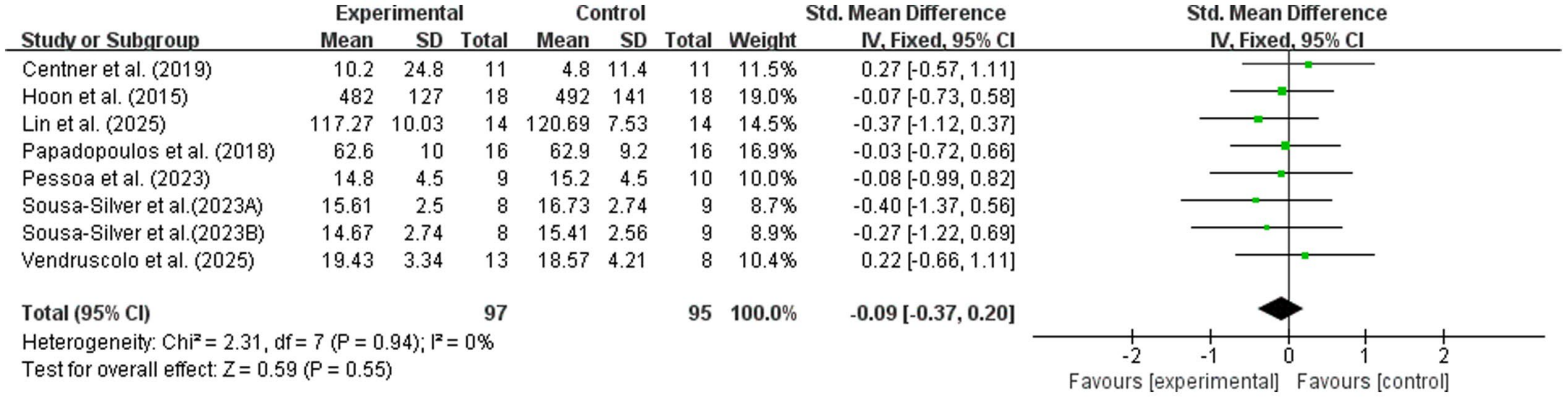
Effect of BFR training combined with nutritional intervention on maximal strength.

Seven studies reported the effects of BFR combined with nutritional interventions on maximal strength (7, 11–15, 17). As shown in Figure 4, the pooled results showed low heterogeneity (*I*^2^ = 0%, *p* = 0.94). The meta-analysis revealed that BFR combined with nutritional intervention did not produce a significant additive effect over BFR alone or BFR with placebo for maximal strength outcomes (SMD = −0.09; 95% CI: −0.37, 0.20; *Z* = 0.59, *p* = 0.55). These findings suggest that, based on the currently available evidence, nutritional supplementation did not significantly enhance strength gains induced by BFR training.

**Figure 5:**
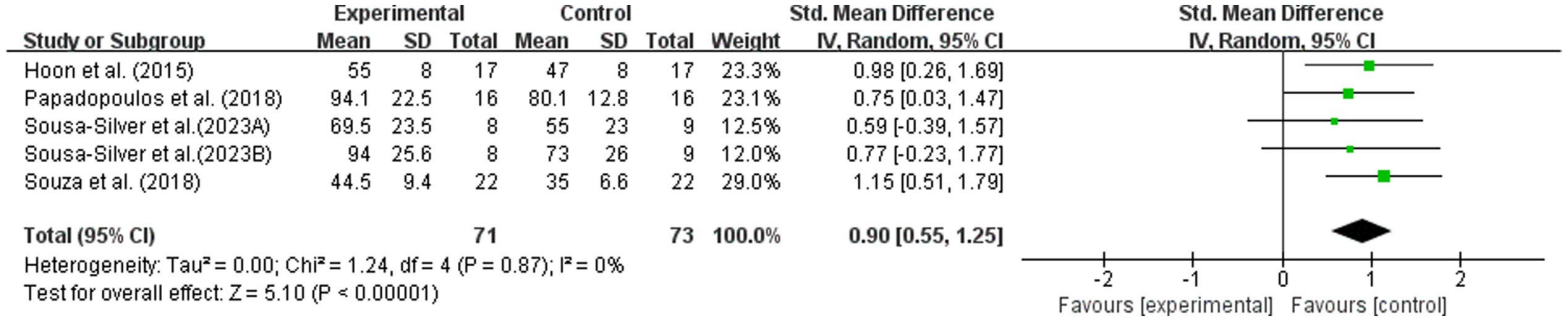
Effect of BFR training combined with nutritional intervention on muscular endurance.

Five studies reported the effects of BFR combined with nutritional interventions on muscular endurance (9, 11, 13, 14, 23). As shown in Figure 5, BFR combined with nutritional intervention significantly improved muscular endurance performance compared with BFR alone or BFR with placebo (SMD = 0.90; 95% CI: 0.55, 1.25; *Z* = 5.10, *p* < 0.00001). The pooled results showed low heterogeneity (*I*^2^ = 0%, *p* = 0.87). This suggests that the observed endurance benefit was relatively consistent across the included studies, although interpretation should remain cautious given the limited number of studies and intervention variability.

**Figure 6:**
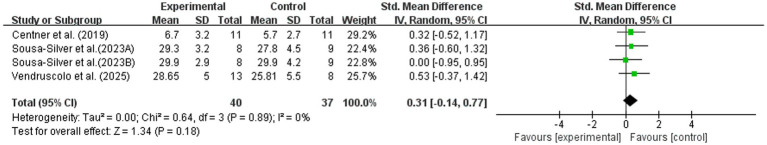
Effect of BFR training combined with nutritional intervention on muscle mass.

Four studies assessed muscle morphology outcomes using MRI or ultrasound, including muscle cross-sectional area (CSA) or muscle thickness (MT) (7, 11, 17). As shown in Figure 6, BFR combined with nutritional intervention did not significantly improve muscle hypertrophy compared with BFR alone or BFR with placebo (SMD = 0.31; 95% CI: −0.14, 0.77; *Z* = 1.34, *p* = 0.18). Low heterogeneity was observed across studies (*I*^2^ = 0%, *p* = 0.89), indicating that the direction of findings was relatively consistent, although the number of included studies was small.

### 3.3 Subgroup analysis

To further explore factors potentially influencing maximal strength outcomes, subgroup analyses were conducted according to nutritional supplement type, participant training status, and intervention duration.

#### 3.3.1 Nutritional supplement type

**Figure 7:**
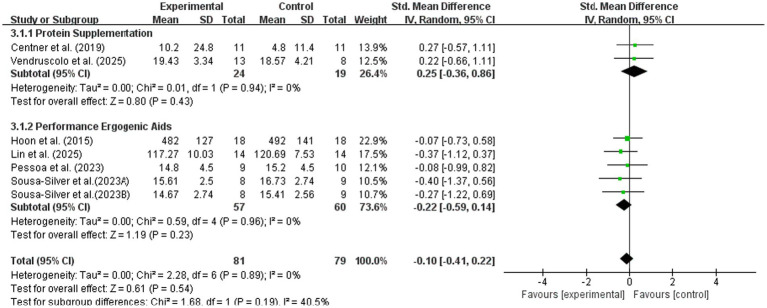
Subgroup analysis of maximal strength by type of nutritional intervention.

Studies were stratified into two subgroups: protein supplementation and functional supplements. As shown in Figure 7, neither subgroup demonstrated a significant improvement in maximal strength compared with the control condition. Protein supplementation showed no significant effect (SMD = 0.25, 95% CI: −0.36, 0.86; *p* = 0.43), and functional supplements likewise showed no significant effect (SMD = −0.22, 95% CI: −0.59, 0.14; *p* = 0.23). No significant subgroup difference was observed (Chi^2^ = 1.68, *p* = 0.19), suggesting that supplement category did not meaningfully modify the strength-related effects of BFR combined with nutrition.

#### 3.3.2 Training status

**Figure 8:**
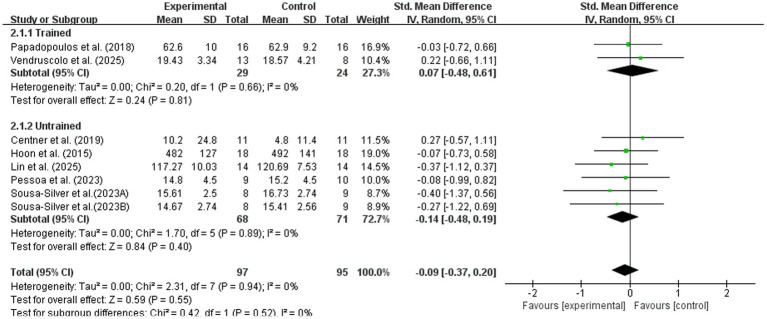
Subgroup analysis of maximal strength by participant characteristics.

Studies were further stratified into trained and untrained subgroups. As shown in Figure 8, neither subgroup demonstrated a significant improvement in maximal strength with BFR combined with nutritional intervention. In the trained subgroup, no significant difference was observed relative to the control group (SMD = 0.07, 95% CI: −0.48, 0.61; *p* = 0.81). Similarly, the untrained subgroup showed no significant effect (SMD = −0.14, 95% CI: −0.48, 0.19; *p* = 0.40). No significant subgroup difference was found (Chi^2^ = 1.68, *p* = 0.52).

#### 3.3.3 Intervention duration

**Figure 9:**
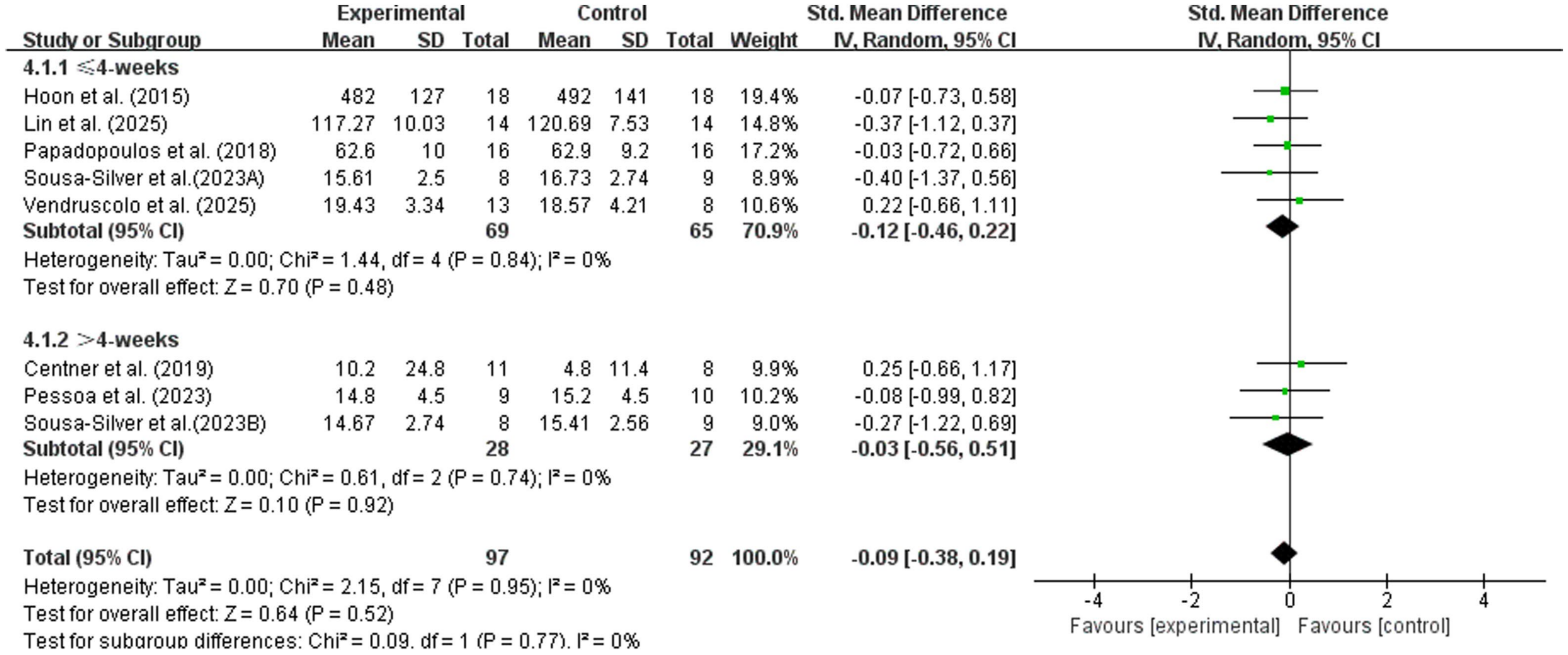
Subgroup analysis of maximal strength by intervention duration.

Studies were divided into short-term/acute and long-term intervention groups. As shown in Figure 9, the intervention duration did not significantly affect the impact of nutritional supplementation on maximal strength with BFR. The short-term group showed SMD = −0.12 (95% CI: −0.46, 0.22; *p* = 0.48), and the long-term group showed SMD = −0.03 (95% CI: −0.56, 0.51; *p* = 0.92). No significant difference was found between the two subgroups (Chi^2^ = 0.09, *p* = 0.77, *I*^2^ = 0%), indicating that the lack of effect of nutritional supplements on maximal strength is not due to insufficient intervention duration.

### 3.4 Publication bias

**Figure 10:**
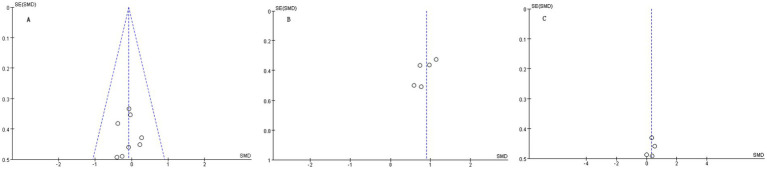
Funnel plot for publication bias.

As shown in Figures 10A–C, funnel plots were visually inspected as an exploratory assessment of publication bias for the pooled outcomes. The plots appeared approximately symmetrical. However, because fewer than 10 studies were available for each pooled analysis, these funnel plots were considered insufficient to formally exclude the possibility of publication bias. Therefore, publication bias cannot be ruled out and the findings should be interpreted with appropriate caution.

## 4 Discussion

To our knowledge, this study is the first systematic review with meta-analysis to assess the combined effects of BFR training and different nutritional interventions on muscle adaptations. Through a comprehensive analysis of nine randomized controlled trials, our main finding indicates that while BFR training combined with nutritional supplementation did not significantly improve maximal strength or muscle hypertrophy, it did show significant and consistent improvements in muscular endurance. This suggests that, under the high metabolic stress environment of BFR training, the main role of nutritional supplements is to alleviate metabolic limitations and delay fatigue, rather than directly promoting structural remodeling.

### 4.1 The effect of nutritional intervention on maximal strength in BFR training

The results of this study indicate that, compared to BFR training alone, combining nutritional interventions did not significantly enhance maximal strength. However, given the small number of included studies and limited sample sizes, these findings should be interpreted cautiously, and the possibility of Type II error cannot be excluded. To explore potential moderating variables, we performed subgroup analyses based on nutritional supplement types, participant characteristics, and intervention durations. However, the analysis consistently showed that neither protein, a structural substrate, nor functional supplements aimed at modulating neuro-metabolism significantly enhanced the increase in maximal strength. This finding likely stems from the potent physiological stimulus induced by BFR training itself. BFR training, by combining low-load resistance with ischemia and hypoxia, induces high metabolic stress in a short period and recruits high-threshold motor units, effectively promoting neuromuscular adaptation (5, 24, 25). In a study by Lin et al. (15), the control group undergoing only 4 weeks of BFR training observed a significant increase in MVC (approximately 20.5%). Similarly, Vendruscolo et al. (17) found that after 3 weeks of BFR training, the 1RM increase in the placebo group was similar to that of the protein supplementation group. This suggests that when the mechanical and metabolic stimuli provided by BFR training approach the physiological adaptation threshold, additional nutritional supplementation does not enhance the effects, and thus, short-term supplementation is unlikely to significantly boost strength gains.

Maximal strength gains primarily depend on neural drive, motor unit synchronization, and the explosive power provided by the phosphagen system. Previous research has demonstrated that high-intensity strength training significantly enhances neural drive, thus improving the ability to produce maximal muscle contractions (26). Although dietary nitrates (such as beetroot juice) can improve skeletal muscle blood flow and enhance microcirculation through NO-mediated vasodilation (27, 28), there is currently insufficient high-quality evidence to show that these improvements in blood flow and metabolism directly translate to significant gains in maximal voluntary contraction (MVC). Papadopoulos et al. (14) found that nitrates improved endurance performance by enhancing microvascular blood flow and calcium ion processing, but did not promote an increase in maximal strength. Recent reviews (25) also indicate that while nitrate supplementation has beneficial effects on endurance performance, it does not exhibit an additive effect on maximal strength. Similarly, although caffeine, a central nervous system stimulant, can enhance neural excitability (29), Lin et al. (15) found that it did not improve MVC under BFR conditions. This may be due to the peripheral metabolic suppression effects induced by BFR training, which could counteract the central excitatory effects of caffeine.

Maximal strength improvements mainly rely on neural drive, motor unit synchronization, and the explosive power from the phosphagen system (30, 31). However, the primary mechanisms of the supplements discussed in this review generally focus on metabolic regulation and fatigue resistance. For example, *β*-alanine mitigates metabolic acidosis (H+) by increasing carnosine content, which primarily delays endurance fatigue but has minimal contribution to single maximal strength that depends on neural recruitment (12, 32). Therefore, despite certain supplements having a positive effect on metabolic processes, they have not significantly enhanced maximal strength gains.

The training status of participants is a key variable affecting the effectiveness of nutritional interventions (33). For individuals with prior training experience, their neuromuscular system has already reached a high level of adaptation, which significantly raises the threshold for further adaptation (17). Consequently, short-term nutritional interventions are unlikely to provide sufficient stimuli to induce significant gains in maximal strength. In contrast, for untrained individuals, early strength gains are primarily driven by neural adaptation (34). This early neural adaptation effect may obscure the minor advantages of nutritional supplements at the metabolic or structural levels. Sousa-Silva et al. (11) support this view: although the creatine group showed a slight advantage at week 4 of training, this difference disappeared by week 8. This suggests that while nutritional supplements may accelerate the adaptation process, they do not alter the long-term peak strength determined by BFR training.

### 4.2 The effect of nutritional intervention on muscle hypertrophy in BFR training

Although protein and creatine are generally considered key nutrients for promoting muscle structural adaptations, the results of this meta-analysis indicate that combined nutritional interventions did not significantly enhance muscle cross-sectional area (CSA) or muscle thickness (MT) under BFR training conditions. This phenomenon may be attributed to the potent anabolic stimulus provided by BFR training itself, which potentially masks the effects of nutritional supplements. BFR training activates the mTORC1 signaling pathway through mechanisms such as metabolic stress, mechanical tension, and cell swelling, thereby promoting muscle protein synthesis (3, 35). Centner et al. (7) found that although the CSA increase in the collagen supplementation group (6.7% vs. 5.7%) was slightly higher than that in the control group, this difference did not reach statistical significance. These results suggest that the strong anabolic stimulus provided by BFR may limit the observable additional effect of nutritional supplementation under the conditions studied.

Furthermore, nutritional supplements may show short-term benefits by accelerating the adaptation process in the early stages of training, but their long-term effects are limited. Sousa-Silva et al. (11) observed that, at week 4, the creatine group showed a slight advantage in muscle thickness compared to the placebo group, which might be attributed to the cell volume increase due to intracellular hydration caused by creatine. However, as the training continued to week 8, the difference in muscle thickness between the BFR and placebo groups disappeared. This suggests that the early advantage of nutritional supplementation may be due to an accelerated morphological change through the increase in non-contractile components. In the long term, the accumulation of myofibrillar proteins caused by BFR training is the decisive factor for muscle hypertrophy.

The training status of the participants and their baseline dietary protein intake levels limit the effectiveness of additional nutritional interventions. Vendruscolo et al. (17) found that extra supplementation with whey protein did not significantly outperform the placebo group in well-trained individuals. The study indicated that these participants already had adequate baseline protein intake (>1.6 g/kg/d), and for trained individuals, a higher mechanical stimulus threshold is required to further promote structural changes. Therefore, BFR training, as a method capable of maximizing the recruitment of high-threshold motor units, becomes the key factor for muscle hypertrophy, while additional nutritional supplementation has limited effects on muscle growth.

### 4.3 The effect of nutritional intervention on muscular endurance in BFR training

Nutritional intervention was associated with a significant improvement in muscular endurance during BFR training. This pattern is physiologically plausible, as BFR creates a metabolically demanding environment characterized by restricted oxygen delivery, metabolite accumulation, and rapid fatigue development. Under such conditions, supplements that influence energy buffering, vascular regulation, or perceptual tolerance may be more likely to affect endurance-related outcomes.

Creatine supplementation may be one possible contributor to the observed endurance benefit. A notable physiological feature of BFR training is the restriction of blood flow during both exercise and inter-set recovery, which may hinder the clearance of metabolic byproducts and impair phosphocreatine (PCr) resynthesis, thereby accelerating fatigue development (36–38). In this context, increased intramuscular PCr availability may help sustain repeated contractions or delay task failure. Sousa-Silva et al. (11) reported a higher number of repetitions to failure at 30% 1RM in the creatine group, which is broadly consistent with this interpretation. However, it should be noted that the included RCTs did not directly assess PCr kinetics, and therefore this explanation remains inferential.

Nitrate supplementation may also represent a plausible explanation for part of the endurance-related effect. Through the nitric oxide (NO) pathway, dietary nitrate may promote vasodilation and improve local perfusion, partially counteracting the ischemic burden imposed by BFR (28). Papadopoulos et al. (14) used near-infrared spectroscopy (NIRS) and found that acute supplementation with nitrate-rich beetroot juice extended time to failure during isometric contractions. In addition, Hoon et al. (13) proposed that nitrate may influence excitation-contraction coupling through calcium-handling mechanisms. Nevertheless, these physiological interpretations should be treated with caution, because they were not consistently or directly verified across all included studies.

A similar caution applies to caffeine. BFR exercise is frequently accompanied by marked discomfort and ischemia-related pain, which may limit endurance performance (5, 39, 40). Caffeine may improve tolerance to such conditions through central nervous system stimulation and reduced perceived discomfort (29). Souza et al. (9) reported that acute caffeine intake increased the total number of repetitions and reduced pain ratings during exercise, suggesting that perceptual modulation may partly explain the endurance-related advantage. However, as with creatine and nitrate, the current evidence does not allow firm mechanistic conclusions, and these explanations should be regarded as plausible rather than confirmed.

Overall, the endurance-related findings appear consistent with the idea that nutritional interventions may be more effective for supporting fatigue resistance than for augmenting maximal strength or hypertrophy under BFR conditions. At the same time, the relatively small number of studies, the diversity of supplements, and the variability in exercise protocols mean that this interpretation remains preliminary.

### 4.4 Limitations and strengths

This study has several limitations that should be acknowledged. First, the number of included studies was relatively small, and the pooled sample size for several outcomes remained limited. Consequently, the meta-analysis may have lacked sufficient statistical power to detect small but potentially meaningful effects, particularly for maximal strength and muscle hypertrophy. Therefore, the non-significant findings in these domains should be interpreted cautiously, and the possibility of Type II error cannot be excluded. Second, although low statistical heterogeneity was observed across pooled analyses, this finding should be interpreted with caution. The low *I*^2^ values may reflect a relatively consistent direction of effects, but they may also be influenced by the small number of studies, limited sample sizes, and the use of similar outcome measures. Accordingly, low heterogeneity should not be taken as definitive evidence of homogeneous physiological responses across all supplementation strategies and populations. Third, the overall risk-of-bias profile was generally acceptable; however, several studies had unclear or insufficient reporting in specific domains, particularly regarding allocation and blinding procedures. This limits the certainty of the current evidence, and the pooled findings should be interpreted as moderate rather than definitive. Fourth, visual inspection of funnel plots did not reveal obvious asymmetry. Nevertheless, because fewer than 10 studies were available for each pooled outcome, publication bias could not be formally evaluated and cannot be ruled out. The apparent symmetry should therefore not be interpreted as evidence that publication bias was absent. Finally, additional limitations include the predominance of male participants (only two studies included females), methodological heterogeneity in BFR cuff pressure protocols and timing of supplementation, and the lack of long-term follow-up (>12 weeks), which limit the generalizability and assessment of sustained effects.

Based on these limitations, future research should focus on: (1) conducting high-quality RCTs including female and elderly sarcopenic populations; (2) investigating molecular and cellular mechanisms of BFR training, such as changes in muscle fiber type and mTOR signaling pathways; and (3) directly comparing the synergistic effects of different dosages and types of supplements to optimize intervention strategies.

## 5 Conclusion

This systematic review with meta-analysis assessed the effects of blood flow restriction (BFR) training combined with nutritional interventions on maximal strength, muscular endurance, and muscle hypertrophy. The current evidence suggests that nutritional interventions do not provide a clear additional benefit for maximal strength or muscle hypertrophy beyond BFR training alone, but may be associated with improvements in muscular endurance. These findings are consistent with the idea that supplementation could help support fatigue resistance under the metabolically demanding conditions induced by BFR training. However, given the limited number of studies and small pooled sample sizes, these conclusions should be interpreted cautiously. Further high-quality research is needed to clarify long-term effects, potential mechanisms, and population-specific responses, including female and elderly participants.

## Data Availability

The original contributions presented in the study are included in the article/supplementary material, further inquiries can be directed to the corresponding author.
