# Supplementary material for: Combined effects of blood flow restriction training and nutritional intervention on muscle adaptations: a systematic review and meta-analysis
Source: Front Nutr. 2026 May 8;13:1762391. doi: 10.3389/fnut.2026.1762391 (PMC13196806; doi:10.3389/fnut.2026.1762391)
Supplement: Supplementary file 1 [file Table_1.docx]

**Literature Search Strategy**

**Table S1.** Search strategy

| **Database** | **Search terms** | **Total Number** |
| --- | --- | --- |
| Pubmed  (2006-2026) | (("blood flow restriction"[Title/Abstract] OR "blood flow restriction training"[Title/Abstract] OR "vascular occlusion"[Title/Abstract] OR "occlusion training"[Title/Abstract] OR "KAATSU"[Title/Abstract]) AND ("nutrition"[Title/Abstract] OR "nutritional intervention"[Title/Abstract] OR "supplement*"[Title/Abstract] OR "protein"[Title/Abstract] OR "whey protein"[Title/Abstract] OR "creatine"[Title/Abstract] OR "caffeine"[Title/Abstract] OR "beta-alanine"[Title/Abstract] OR "β-alanine"[Title/Abstract] OR "nitrate"[Title/Abstract] OR "beetroot juice"[Title/Abstract] OR "vitamin*"[Title/Abstract] OR "collagen"[Title/Abstract] OR "betaine"[Title/Abstract]) AND ("strength"[Title/Abstract] OR "maximal strength"[Title/Abstract] OR "one-repetition maximum"[Title/Abstract] OR "1RM"[Title/Abstract] OR "maximal voluntary contraction"[Title/Abstract] OR "MVC"[Title/Abstract] OR "muscular endurance"[Title/Abstract] OR "endurance"[Title/Abstract] OR "fatigue resistance"[Title/Abstract] OR "time to fatigue"[Title/Abstract] OR "repetitions to failure"[Title/Abstract] OR "muscle hypertrophy"[Title/Abstract] OR "muscle thickness"[Title/Abstract] OR "cross-sectional area"[Title/Abstract] OR "CSA"[Title/Abstract] OR "fat-free mass"[Title/Abstract] OR "FFM"[Title/Abstract])) | 168 |
| Web of Science | TS=(("blood flow restriction" OR "blood flow restriction training" OR "vascular occlusion" OR "occlusion training" OR "KAATSU") AND ("nutrition" OR "nutritional intervention" OR "supplement*" OR "protein" OR "whey protein" OR "creatine" OR "caffeine" OR "beta-alanine" OR "β-alanine" OR "nitrate" OR "beetroot juice" OR "vitamin*" OR "collagen" OR "betaine") AND ("strength" OR "maximal strength" OR "one-repetition maximum" OR "1RM" OR "maximal voluntary contraction" OR "MVC" OR "muscular endurance" OR "endurance" OR "fatigue resistance" OR "time to fatigue" OR "repetitions to failure" OR "muscle hypertrophy" OR "muscle thickness" OR "cross-sectional area" OR "CSA" OR "fat-free mass" OR "FFM")) | 211 |
| Scopus | TITLE-ABS-KEY (("blood flow restriction" OR "blood flow restriction training" OR "vascular occlusion" OR "occlusion training" OR "KAATSU")) AND TITLE-ABS-KEY (("nutrition" OR "nutritional intervention" OR "supplement*" OR "protein" OR "whey protein" OR "creatine" OR "caffeine" OR "beta-alanine" OR "β-alanine" OR "nitrate" OR "beetroot juice" OR "vitamin*" OR "collagen" OR "betaine")) AND TITLE-ABS-KEY (("strength" OR "maximal strength" OR "one-repetition maximum" OR "1RM" OR "maximal voluntary contraction" OR "MVC" OR "muscular endurance" OR "endurance" OR "fatigue resistance" OR "time to fatigue" OR "repetitions to failure" OR "muscle hypertrophy" OR "muscle thickness" OR "cross-sectional area" OR "CSA" OR "fat-free mass" OR "FFM")) | 236 |
| Embase | ('blood flow restriction':ti,ab,kw OR 'blood flow restriction training':ti,ab,kw OR 'vascular occlusion':ti,ab,kw OR 'occlusion training':ti,ab,kw OR 'KAATSU':ti,ab,kw) AND ('nutrition':ti,ab,kw OR 'nutritional intervention':ti,ab,kw OR 'supplement*':ti,ab,kw OR 'protein':ti,ab,kw OR 'whey protein':ti,ab,kw OR 'creatine':ti,ab,kw OR 'caffeine':ti,ab,kw OR 'beta-alanine':ti,ab,kw OR 'β-alanine':ti,ab,kw OR 'nitrate':ti,ab,kw OR 'beetroot juice':ti,ab,kw OR 'vitamin*':ti,ab,kw OR 'collagen':ti,ab,kw OR 'betaine':ti,ab,kw) AND ('strength':ti,ab,kw OR 'maximal strength':ti,ab,kw OR 'one-repetition maximum':ti,ab,kw OR '1RM':ti,ab,kw OR 'maximal voluntary contraction':ti,ab,kw OR 'MVC':ti,ab,kw OR 'muscular endurance':ti,ab,kw OR 'endurance':ti,ab,kw OR 'fatigue resistance':ti,ab,kw OR 'time to fatigue':ti,ab,kw OR 'repetitions to failure':ti,ab,kw OR 'muscle hypertrophy':ti,ab,kw OR 'muscle thickness':ti,ab,kw OR 'cross-sectional area':ti,ab,kw OR 'CSA':ti,ab,kw OR 'fat-free mass':ti,ab,kw OR 'FFM':ti,ab,kw) | 198 |
| Cochrane Library | ("blood flow restriction" OR "blood flow restriction training" OR "vascular occlusion" OR "occlusion training" OR "KAATSU"):ti,ab,kw AND ("nutrition" OR "nutritional intervention" OR "supplement*" OR "protein" OR "whey protein" OR "creatine" OR "caffeine" OR "beta-alanine" OR "β-alanine" OR "nitrate" OR "beetroot juice" OR "vitamin*" OR "collagen" OR "betaine"):ti,ab,kw AND ("strength" OR "maximal strength" OR "one-repetition maximum" OR "1RM" OR "maximal voluntary contraction" OR "MVC" OR "muscular endurance" OR "endurance" OR "fatigue resistance" OR "time to fatigue" OR "repetitions to failure" OR "muscle hypertrophy" OR "muscle thickness" OR "cross-sectional area" OR "CSA" OR "fat-free mass" OR "FFM"):ti,ab,kw | 45 |

Abbreviations: BFR, blood flow restriction; MVC, maximal voluntary contraction; 1RM, one-repetition maximum; CSA, cross-sectional area; FFM, fat-free mass.
